# Using a new, innovative “mixed reality” technology in endoscopic retrograde cholangiopancreatography

**DOI:** 10.1055/a-2421-6161

**Published:** 2024-10-25

**Authors:** Yuki Ishikawa-Kakiya, Hirotsugu Maruyama, Kojiro Tanoue, Akira Higashimori, Shusei Fukunaga, Yasuhiro Fujiwara

**Affiliations:** 1 Department of Gastroenterology, Osaka Metropolitan University Graduate School of Medicine, Osaka, Japan

An in-depth understanding of a patient’s anatomy is important for endoscopic retrograde cholangiopancreatography (ERCP). Computed tomography (CT) and magnetic resonance cholangiopancreatography (MRCP) provide two-dimensional images, individually converted into three-dimensional (3D) head images. Trainees may find it difficult to understand the 3D anatomical images of the pancreaticobiliary region by CT and/or MRCP and may not match the trainer’s image. Innovative technology, known as “mixed reality,” which combines virtual reality and the real world, is used medically 1 2 3 . Mixed reality enables the display of 3D holograms in real space and aids users to confirm the stereoscopic anatomy. Mixed reality has never been used in the endoscopic field. We report a case of ERCP performed using a mixed reality navigation system.

The patient was an 80-year-old woman with suspected congenital biliary dilatation Todani classification type III confirmed by MRCP ( Fig. 1 ). Endoscopic ultrasonography showed a nodule in the choledochal cyst ( Fig. 2 ), and ERCP was planned for cytological assessment. We constructed 3D models of the liver, portal vein, hepatic artery, and bile duct by contrast-enhanced CT and MRCP using SYNAPSE VINCENT (FujiFilm Medical Co., Ltd., Tokyo, Japan). The models were uploaded in the Holoeyes MD system (Holoeyes Inc., Tokyo, Japan) and visualized using “HoloLens 2” goggles (Microsoft Corporation, Redmond, Washington, USA), enabling simultaneous observation of the real world along with the projected 3D hologram ( Fig. 3 , Video 1 ). We verified this from various angles by holding the hologram and checking it with the 3D hologram and fluoroscopic images at the same time ( Fig. 4 ). The assistant collaboratively viewed the same hologram ( Fig. 5 ). There were no adverse events with the goggles. The endoscopist and assistant could confirm the same 3D hologram in real time, which is useful for understanding and educational purposes during and after ERCP. We discovered the possibilities of an innovative mixed reality navigation system in the pancreaticobiliary endoscopy field.

**Fig. 1:**
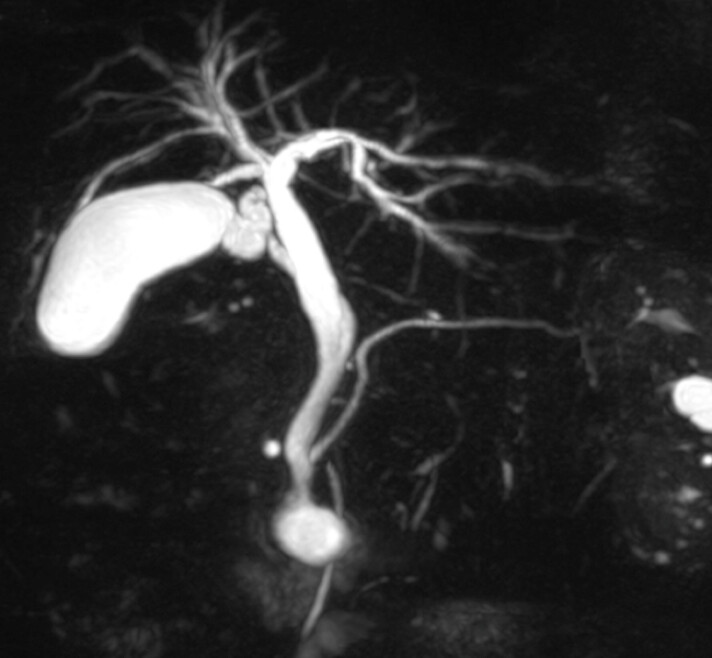
Magnetic resonance cholangiopancreatography showed congenital biliary dilatation.

**Fig. 2:**
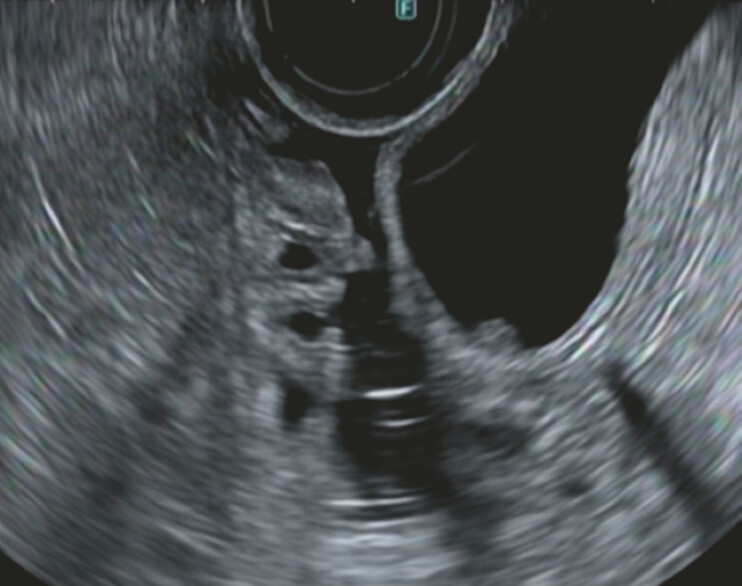
Endoscopic ultrasonography showed nodules in the choledochal cyst.

**Fig. 3:**
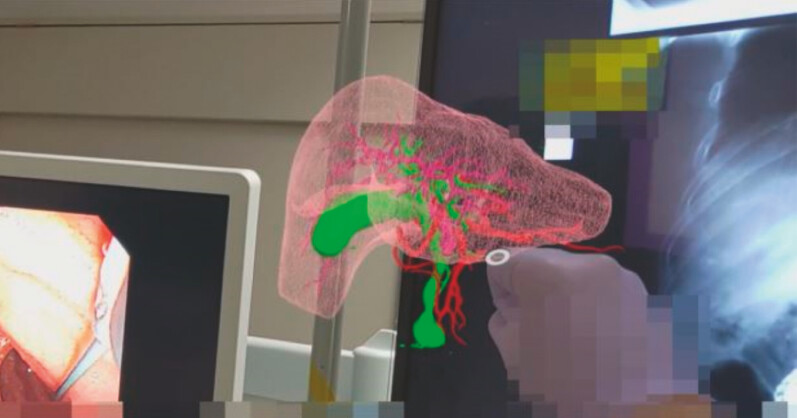
Three-dimensional hologram was visualized using “HoloLens 2” goggles and enabled simultaneous observation of the real world along with the projected 3D hologram.

**Fig. 4:**
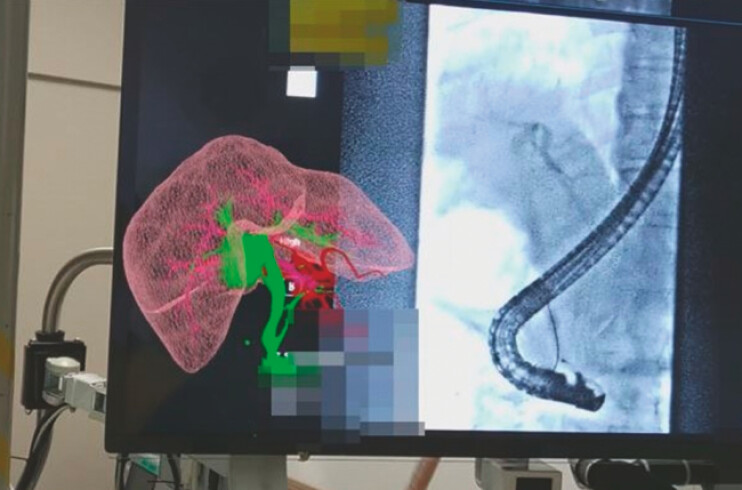
We verified the 3D hologram from various angles and checked it with the fluoroscopic images at the same time.

**Fig. 5:**
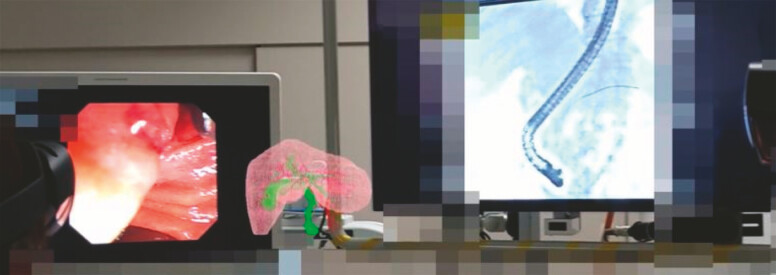
The endoscopist and the assistant collaboratively viewed the same 3D hologram.

Mixed reality navigation with endoscopic retrograde cholangiopancreatography.Video 1

Endoscopy_UCTN_Code_TTT_1AR_2AB

## Notes

Endoscopy E-Videos https://eref.thieme.de/e-videos
*E-Videos* is an open access online section of the journal *Endoscopy* , reporting on interesting cases and new techniques in gastroenterological endoscopy. All papers include a high-quality video and are published with a Creative Commons CC-BY license. Endoscopy E-Videos qualify for HINARI discounts and waivers and eligibility is automatically checked during the submission process. We grant 100% waivers to articles whose corresponding authors are based in Group A countries and 50% waivers to those who are based in Group B countries as classified by Research4Life (see: https://www.research4life.org/access/eligibility/ ). This section has its own submission website at https://mc.manuscriptcentral.com/e-videos .
